# Teacher questions and student responses in case-based learning: outcomes of a video study in medical education

**DOI:** 10.1186/s12909-019-1895-1

**Published:** 2019-12-05

**Authors:** Martin Gartmeier, Theresa Pfurtscheller, Alexander Hapfelmeier, Marc Grünewald, Janina Häusler, Tina Seidel, Pascal O. Berberat

**Affiliations:** 1 Technical University of Munich, TUM School of Medicine, TUM Medical Education Center, Munich, Germany; 2 Institute of Medical Informatics, Statistics and Epidemiology, TUM School of Medicine, Technical University of Munich, Munich, Germany; 3 TUM School of Education, Friedl Schöller Endowed Chair for Educational Psychology, Technical University of Munich, Munich, Germany

**Keywords:** Case-based learning, Teacher questions, Teaching methods, Video study, Student elaboration

## Abstract

**Background:**

Case-based learning (CBL) is a highly interactive instructional format widely used in medical education. One goal of CBL is to integrate basic biomedical knowledge and its application to concrete patient cases and their clinical management. In this context, we focus the role of teacher questions as triggers for reproductive vs. elaborative student responses. Specifically, our research questions concern the kinds of questions posed by clinical teachers, the kinds of responses given by students, the prediction of student responses based upon teacher questions, and the differences between the two medical disciplines in focus of our study, internal medicine and surgery.

**Methods:**

We analyse 19 videotaped seminars (nine internal medicine, ten surgery) taught by clinicians and attended by advanced medical students. Multiple raters performed a low-inference rating process using a theory-based categorical scheme with satisfactory interrater-reliability.

**Results:**

We found that medical teachers mostly posed initial (instead of follow-up) questions and that their questions were more often closed (instead of open). Also, more reasoning (than reproductive) questions were posed. A high rate of student non-response was observed while elaborative and reproductive student responses had a similar prevalence. In the prediction context, follow-up reasoning questions were associated with low non-response and many elaborative answers. In contrast, the highest student non-response rate followed open reproduction questions and initial reasoning questions. Most reproductive statements by students were made following closed reproduction questions.

**Conclusions:**

These results deepen our understanding of interactive, questions-driven medical teaching and provide an empirical basis for clinical teachers to use questions in didactically fruitful ways.

## Background

The application of basic biomedical knowledge to specific patient cases is a core element of medicine, as a discipline and practice. Instructional approaches such as case-based learning (CBL) have hence become essential elements of many medical curricula and they represent “fundamental ways in which future practitioners are educated for their new profession” ([1], p. 52). Despite many different forms and didactic designs of CBL existing [2–5], a core element of this format is a teacher-guided discussion of a patient case during which students “collaboratively apply learned principles and data analyses and evaluate the usefulness of various strategies to achieve optimal resolutions for the problems posed” ([6], p. 835). This means that CBL is a highly interactive seminar format in various respects: First, an experienced physician guides students through a clinical case while activating their basic knowledge and engaging them in clinical reasoning processes, mainly through asking questions. These questions may be dedicated to clarify students understanding of different pathological phenomena, to the clinical management of specific patients and to therapeutic consequences of diagnostic evidence [4, 7]. Second, through the answers students give and through the questions they pose themselves, students can actively influence the way in which a clinical case is discussed and analysed [6, 8–11]. Third, clinical teachers also use peer-learning methods (especially small group discussions [12];) as highly interactive didactic elements. This means that for some periods of time during the seminar, students form groups, e.g. to discuss and make sense of outcomes and consequences of diagnostic procedures.

Given the interactive nature of CBL, it is remarkable that empirical evidence from medical education about teacher-student interaction patterns characterizing this instructional format is still rare ([13] report a relevant study from the nursing context as a related professional field). Instead, existing studies on CBL often focus on structural surface aspects (such as type and number of cases or length of exposure to cases, cf. [4]) or on outcomes of this method [14–18]. We propose that to better understand teaching and learning in CBL, it should be conceptualized as a form of *dialogic teaching*. This thriving strand of educational research capitalises upon the idea that didactically orchestrated classroom discussion is a contemporary and powerful pedagogic strategy [19, 20]. In the present study, we focus upon *teachers’ question asking* and *students’ responding and non-responding behaviours* as didactically essential components of case-based pedagogies. The study is a low-inference video study (e.g., [21–24]) in which we analyse the interaction between teachers and students in a sample of video-recorded case-based seminars from internal medicine and surgery. In the following, we will clarify the instructional value of teacher questions and their association with reproductive vs. elaborative student responses in the context of CBL.

### Teacher questions as predictors of student responses in CBL

As CBL is an essentially learner-centred format [4], teacher questions are only a means to an end in this context. The dependent variable of our study are student responses and we analyse whether these have *reproductive* vs. *elaborative* quality (cf. categories 4.3 and 4.4 in Table 1). The key point of CBL is to confront students with “a dialectic of the general with the particular” ([1], p.52), meaning that basic knowledge is supposed to be *activated* and *applied* to specific cases. In this sense, we differentiate *reproductive* and *elaborative student statements* in the present study*.* Reproductive statements focus upon basic biomedical knowledge which advanced medical students should have, e.g., from textbooks or from pre-clinical medical education. *Elaborative statements* are made when basic knowledge is applied to specific patient cases, e.g. when students engage in considerations and clinical reasoning regarding the concrete case discussed in the seminar. A similar distinction has been made by Chéron, Ademi, Kraft, and Löffler-Stastka [25] who differentiated two types of multiple choice questions students created in CBL – *knowledge recall items* (testing basic science knowledge) and *application of knowledge items* (anchored in patient vignettes). Elaborative statements also indicate what has been labelled as *deep reasoning* in context of another video study [26]. Deep reasoning activities comprise explanations of if-then and cause-effect relationships and require students to combine and evaluate information from various sources and to make predictions about how specific situations will develop given the presence of particular influential factors. Finally, reproductive and elaborative responses can be related to the revised form of Bloom’s taxonomy [27]: Reproductive questions basically demand *remembering* information, whereas elaborative questions demand *applying* knowledge to cases in order to *analyse*/*evaluate* (specific aspects of) these cases.

**Table 1:** Categories of teacher questions and student responses

Coding scheme	Values	Brief description	Example from the coded material
Teacher questions			
Didactically irrelevant teacher questions (unrelated to the learning goals of the seminar)			
0. Didactically irrelevant questions	0.1 Organizational questions	… are focused upon aspects of seminar organization	“Do you need my signature to confirm you have attended the seminar?”
	0.2 Comprehension questions	… contain a request directed towards a student to repeat a statement which the teacher hasn’t understood properly (e.g. due to noise in the classroom).	“I somehow didn’t hear you. Can you please say that again?”
Didactically relevant questions (related to the learning goals of the seminar)			
1. Type of questions	1.1 Initial Questions	… are teacher questions related to the case discussed in the seminar. These questions do not build upon a question posed right before.	“Which diagnostic procedures would you apply at this point?”
	1.2 Follow-up questions	… build upon a previous student response and/or an initial question	*(Relating to the previous example)* “In which sequence would you apply these diagnostic procedures?”
2. Openness of questions	2.1 Open questions	… are not supposed to elicit one specific correct answer from students. They provide degrees of freedom to students to elaborate their thoughts.	“Do we already have all the information we need or is there anything we still need to know?”
	2.2 Closed questions	… have only one (or very few) correct answers the teacher intends to elicit from students.	“If you examine a patients’ x-ray image – what is the first thing you need to look at?”
3. Cognitive level of questions	3.1 Reproduction questions	… focus upon information students should be familiar with (e.g. from previous courses) and need to be remembered to answer the question.	“What is the MCH value, what does it tell you?”
	3.2 Elaboration questions	… require combining, interpreting, or weighing information to elaborate and explain issues.	“Can your elaborate which diagnostic strategy makes more sense at this point?”
Student responses to teacher questions			
4. Type of student responses	4.1 Organizational statements	… concern aspects of seminar organization	*(In response to teacher question exemplified above, category 0.1)* “Yes, we need your signature on this form here.”
	4.2 Questions	… posed in response to a teacher question. (Questions which are related to organizational aspects are coded as 4.1.)	“Do I know the history of the patient?”
	4.3 Reproductive statements	… are contributions in which basic knowledge (e.g., from pre-clinical medical education) is reiterated (facts, numbers, or brief explanations).	*(In response to teacher question exemplified above, category 3.1)* “MCH means mean corpuscular haemoglobin, I think it’s about how much haemoglobin is in a red blood cell.”
	4.4 Elaboration / Reasoning statements	… describe cause-effect or if-then relationships and considerations relevant for clinical decision making.	“One reason for constant thirst could be excretion problems. We should check the respective lab parameters to see whether his kidneys work properly.”
	4.5 Non-response	No student answer to a teacher question is given	

In fact, the investigation of teacher questions in interactive, student centred instructional formats is a thriving issue of empirical education, especially school-related research. In a study on lower secondary school teachers, Sedova, Sedlacek, and Svaricek [28] show that the use of open-ended teacher questions with high cognitive demand is positively associated with high-quality classroom discourse. Chinn, Anderson, and Waggoner [29] trained language teachers in *collaborative reasoning*, a student-centred teaching format characterized by facilitative teacher questions. Their study showed that in collaborative reasoning discussions, students not only showed higher engagement, but also more often used higher order cognitive processes. These results are in line with outcomes of a meta-analysis by Redfield and Rousseau [30]. Summarizing twenty studies on the effects of teacher questions with various cognitive demands, gains in achievement-related outcomes were associated with questions that posed higher cognitive demands upon students.

### Focus of the study

Regarding clinical teachers, asking questions has been earlier described as a key strategy to engage students in CBL [31, 32]. Questions are useful to probe students’ basic knowledge and guide them to apply this knowledge in the complex decisions and considerations involved in managing a patient case. So, the aim of our study is to shed light upon how effective different kinds of questions posed by clinical teachers are in eliciting basic biomedical knowledge from students (reproduction) and make them apply this knowledge to a concrete case (elaboration). We thereby focus upon three qualities of teacher questions, i.e., whether they are open or closed, whether they have initial or follow-up character and whether they are reproductive or elaborative in nature. Besides this focus, the present study also explores how CBL is practised in two medical disciplines: internal medicine and surgery. In doing so, we adopt a post-positivist epistemological approach [33]: We use (quantitative and qualitative) empirical methods as a means to detect relationships in our data which we seek to generalize beyond the specific sample. In summary, the following research questions are posed:

1. Which types of questions do clinical teachers pose in CBL?
2. How do students respond to teacher questions in CBL seminars?
3. How does the type of questions asked by clinical teachers predict reproductive / elaborative responses and non-response by students in CBL?
4. Are there differences in the questions asked and the answers given between internal medicine and surgery?

## Methods

### Sample: case based seminars

We videotaped 32 case-based clinical seminars (16 internal medicine, 16 surgery). We did not include seminars from other medical fields as in the local medical curriculum, this specific seminar format is only offered in these two disciplines. These, however, include several subdisciplines (see following section). Altogether, about 72 seminars of this type take place each semester at the Technical University of Munich (TUM). Each medical student has to attend three such seminars in the second clinical year. Each seminar features a patient case from the respective discipline being discussed in detail in the session. The cases are not interconnected among each other. All clinical teachers are practicing physicians. They are instructed to focus the application of basic biomedical knowledge in clinical practice in the seminars, to work out a diagnostic and therapeutic rationale for the case together with students and to highlight the relevance of applying the SOAP-scheme for this purpose (e.g., [34]). The lecturers were advised to structure their lectures along the following topics: initial patient case presentation, initial physical examination, discussion of findings, generation of working hypothesis and differential diagnoses, diagnostic measures (laboratory, radiology, MRT, EKG, etc.) and interpretation of results, diagnostic and therapeutic consequences, follow-up and case summary. Some teachers included several mini-lectures in which they presented background information, some included phases of small-group work, e.g. for students to review and interpret results of diagnostic procedures. However, the dominant didactic form across all filmed seminars was a teacher-guided whole group discussion of the patient case along the topics detailed above. The 32 videotaped seminars were taught by 21 different teachers. This means we filmed some teachers several times (two teachers were filmed five times and three teachers were filmed two times). From these teachers, we used only the videos we had filmed first to avoid bias by overweighting individual teachers in the present analysis. We had to exclude three videos due to technical problems. Unfortunately, two of these three videos were from a teacher we had filmed only once. So, we could not analyse seminars from all 21, but only from 19 different teachers (nine internal medicine, ten surgery). Officially, the seminars were supposed to last 120 min. The duration was measured from when the teacher addressed the audience to open the seminar until the seminar was officially closed by the teacher. The average duration of a seminar was 83 min (*Min* = 62, *Max* = 104, *SD* = 10.89).

### Sample: study participants and teacher questions

On average, the teachers in the present sample were 38 years old (*SD* = 6.25; *Min* = 31; *Max* = 57). Their work experience ranged from *Min* = 4 to *Max* = 28 years, the median was 7 years. Three teachers were female and 16 were male. Within the two general subject areas, internal medicine and surgery, teachers from several subdisciplines were in our sample: internal medicine comprised oncology, nephrology, and hematooncology. Surgery comprised trauma, oral and maxillofacial, plastic, vascular, and orthopaedic surgery. All teachers taught cases from their own medical specialization. The average number of students in the seminars was 15 (*SD* = 2.48, Min = 10, Max = 20). On average, the students were in the 8th semester of their medical studies (*SD* = 0.94) and were 24 years old (*SD* = 2.91). The primary unit of analysis in the present study are teacher questions and subsequent student answers or missing answers, respectively. Overall, we observed and analysed a total amount of 1688 teacher questions and events following these questions (including student responses and non-response events).

### Video-analytic research method

**Fig. 1:**
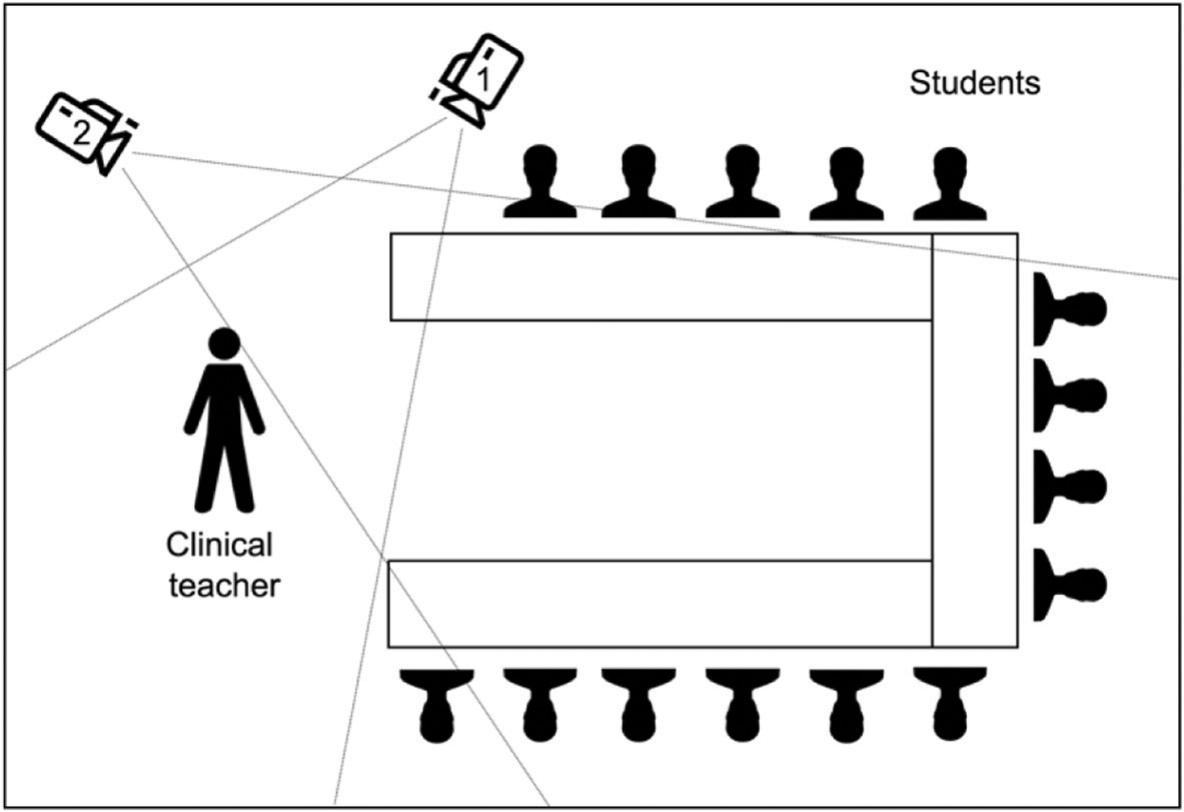
Seminar room and camera setup during the video recording procedure. 1 = Camera one; 2 = Camera two

The present study follows a video-analytic methodological approach adopted from empirical educational (especially school-focused) research (e.g., [21–24]). In brief, studies in this research tradition rely upon video recordings of teaching events to fully capture the interaction between lecturers and learners [26]. The collected video data are analysed by trained raters using a theory-based categorical scheme while monitoring interrater reliability. To perform the video analyses, specific software is used which allows for direct coding of the video material (no transcripts required). This analytic step is primarily qualitative in nature as categories are assigned to events in the videos. From this process, however, emerge quantitative data on the frequency and quality of specific teaching and learning behaviours (in the present case, on questioning and responding to questions). In this way, theory-based quality criteria formulated for specific instructional formats (such as CBL) can be operationalized and reliably observed as a basis for empirical analysis. For collecting the video material we followed a standardised procedure adopted from Seidel et al. [26]. The recordings were made by trained staff from our research team using two cameras (cf. Fig. 1): camera one followed the clinical teacher when moving around the classroom; camera two was an overview camera directed towards the audience.

### Coding process

Coding was done by four researchers (MaGa, MaGr, JaHä, TePf) from our group using the software *Mangold Interact* [35]. In round one, we coded *who was speaking* (teacher / student / several students / nobody / other person) and *what kind of utterance was made* by students (reproductive statement / elaborative statement / content-related question / other statement) and by teachers (giving a short response, explaining facts or clarifying terms / asking a question / explaining subject matter / providing help / giving an instruction or explaining a task / giving examples / summarizing / calling up / giving feedback / clarifying organizational matters). This means we segmented the entire video into consecutive time intervals of varying length representing specific activities by teachers and students and breaks which occurred between these activities. In coding round two, we further categorized these activities as is elaborated in the following section *categorical scheme* (whereby we only specify the codes relevant in the present study). For an initial training phase, two videos of case-based seminars were coded by all four raters. These videos had been recorded in a pilot study and were not part of sample for this study. We calculated interrater agreement between pairs of raters and simultaneously visualised different raters’ coding solutions. On this basis, passages in the seminars in which differences between coders had emerged could be identified easily. These passages were discussed, differences in agreement were resolved, and coding rules refined. This was done in an iterative manner until overall interrater agreement between all four coders, measured by Cohen’s Kappa, reached a satisfactory value of .80 (pairwise values were between .76 and .83). The main study videos were divided between the four raters. Two of the main study videos were analysed by all four coders, resulting in an overall IRR-value of Cohen’s Kappa = 0.65 and pairwise values between .59 and .66, which can be regarded as satisfactory [36].

### Categorical scheme

We analysed the video material using a hierarchical categorical scheme which was based upon published rating schemes [26, 37]. The first focus of analysis are teacher statements coded as questions. We first differentiated whether questions were posed with didactic intention or not. Questions with no didactic relevance were organizational or comprehension-related (Coding Scheme 0, categories 0.1 and 0.2 in Table 1). The latter were posed by clinical teachers in cases when they had not properly understood a student statement. By definition, these *didactically irrelevant teacher questions* are unrelated to the learning goals of the seminar and were hence not considered in the further analyses. *Didactically relevant teacher questions* were further described by the coding schemes *type of question*, *openness of question,* and *cognitive level of question* [26]. Regarding *type of question*, we differentiated *initial* and *follow-up* questions (Categorical Scheme 1 in Table 1). Initial questions are posed by clinical teachers to engage students in a new perspective on a specific topic, they do not build upon previous questions. In contrast, follow-up questions do so, they are asked to deepen, elaborate, or differentiate subject matter [38]. Then, regarding *openness of questions*, we differentiated *closed* and *open questions* (Categorical Scheme 2 in Table 1). Closed questions are intended to elicit one specific correct answer – often only one single term, sometimes a brief list of terms [39]. Open questions are not posed to elicit a specific correct answer, but to stimulate students to verbalize their thoughts and reflections. They invite more differentiated, deliberative answers and give more degrees of freedom to students [38]. Finally, we focused *cognitive level of teacher questions* (Coding Scheme 3 in Table 1) differentiating *reproduction* and *elaboration (or reasoning) questions*. The former are intended to elicit information that should already be known to students, e.g. from pre-clinical medical education. The latter are asked to elucidate cause-effect relationships, differentiate intended from unintended consequences of therapeutic measures, or compare advantages and disadvantages of different therapies and their relevance for the particular case at hand.

Further, we coded different types of student statements made in response to teacher questions. The main categories here were reproductive / elaborative statements and non-response (categories 4.3, 4.4, 4.5 in Table 1). Moreover, students reacted to teacher questions by making organizational statements or by asking questions (code 4.1 and 4.2 in Table 1). The latter codes were, however, not considered in the further analyses. For all categorical Schemes 0–4, teacher or student utterances that could not be assigned were placed into a residual category, *other*. As this was the case in a very small number of instances, we excluded these utterances from all further analyses (cf. results section).

### Statistical analyses

After exporting the codes and performing several steps of data editing, one file was generated which contained data from all seminars. In this file, the time-codes were brought into the correct format so numerical transformations and calculations could be performed. In the following, data is presented by absolute and relative frequencies, distributions are described by median, range, and interquartile range (IQR). Boxplots are used to display the distribution of the relative frequencies of the categorised question characteristics within seminars. Corresponding hypothesis testing on the differences between seminars from internal medicine and surgery was performed by two-sided Mann-Whitney U tests on exploratory 5% significance levels. Teacher questions were investigated as predictors of student answers by evolutionary learning of globally optimal classification trees [40]. This machine learning method is particularly suitable for the present research question as it defines decision rules that lead to an optimal partition of a modelled outcome. In this way, it was possible to perform inherent variable selection and to identify relevant combinations of question characteristics leading to differences in student answers. The hierarchical structure of the decision trees also supports the identification of interactions, i.e., relevant combinations of characteristics of questions.

### Ethical considerations

The current study was approved by the ethics committee of the TUM Rechts der Isar University Hospital (Application code 400/16 S). All teachers and students were informed about the study prior to the seminar via email. One physician refused to take part, so we did not record that respective seminar. At the beginning of each seminar (before starting the video recording), one person from the research team was present to inform all participants about the study, answer open questions, and obtain consent in written from all individuals. We started the video recording only after consent had been declared by all individuals in the room. In one seminar, one student declared unwillingness to be filmed. The student was offered to be seated right beside camera one (cf. Fig. 1), so the student was only audible, but not visible from the viewpoints of both cameras and could fully participate in the seminar. In the further analyses, we included the respective videos, but not the responses given by the specific student.

## Results

Subsequently, we report descriptive results on teacher questions and student responses observed in the CBL seminars.

Table 2 shows the amount of teacher questions posed and student responses given per seminar and per minute. Regarding the two medical disciplines, surgical teachers asked more questions than their colleagues from internal medicine (*p* = 0.81 for absolute numbers and *p* = 0.94 for questions per minute). In contrast, students made more responses in internal medicine (*p* = 0.17 for absolute numbers and *p* = 0.12 for questions per minute).

**Table 2:** Frequencies of teacher questions and student responses per discipline (total and per minute)

	*MD*	*Min*	*Max*	IQR
Teacher questions per seminar				
Overall	98	47	205	80–121
Internal medicine	95	72	205	74–130
Surgery	100	47	203	86–112
Teacher questions per minute				
Overall	1.15	0.68	2.29	0.95–1.72
Internal medicine	1.11	0.80	2.29	0.85–1.97
Surgery	1.15	0.68	1.99	0.99–1.37
Student responses per seminar				
Overall	112	54	234	86.5–164
Internal medicine	137	85	234	100–168
Surgery	94.5	54	220	61–145
Student responses per minute				
Overall	1.26	0.74	2.59	0.94–2.00
Internal medicine	1.87	0.92	2.59	1.16–2.13
Surgery	1.16	0.74	2.13	0.79–1.56

### RQ 1: Which types of questions do clinical teachers pose in CBL?

**Fig. 2:**
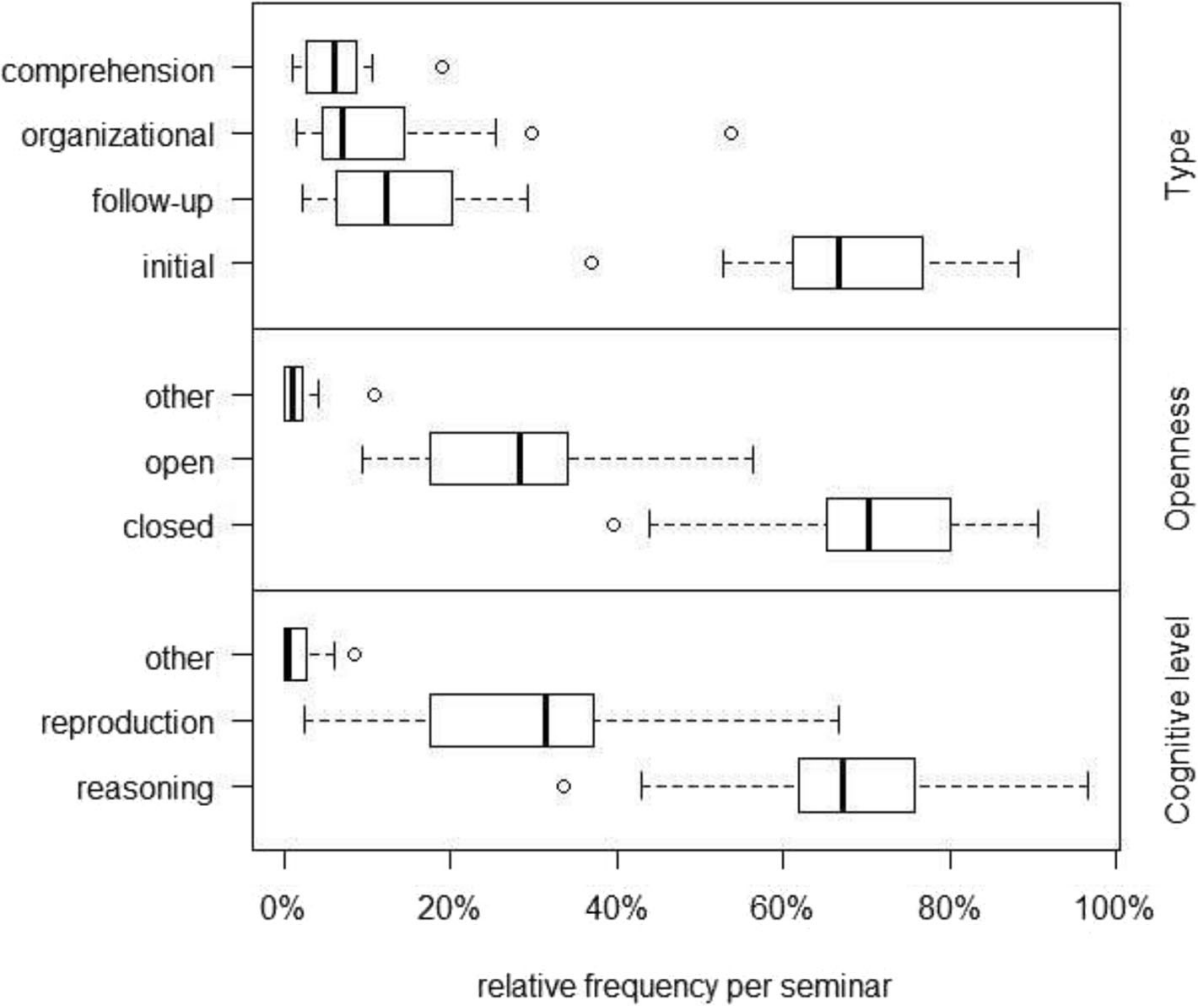
Relative frequencies of different qualities of teacher questions as percentages of the total amount of questions posed per seminar *(“Type” includes didactically irrelevant questions and type of questions)*. Categories add up to 100% in all three sections Type, Openness and Cognitive level. Boxplots show median (MD), minimum (Min), maximum (Max), and interquartile range (IQR) of distributions [41]

To answer this question, we describe the prevalence of the different qualities of teacher questions in our video data. Each single teacher question is characterized by a combination of categories, hence a separate analysis of the prevalence of different combinations is reported below. Figure 2 visualizes the prevalence of different qualities of questions.

As was expected, few of the questions posed by clinical teachers were simply focused upon comprehension of student statements (*MD* = 6%) or were organizational questions (*MD* = 7%). However, there were outliers in organizational questions, indicating a relatively high prevalence (25% up to almost 51%) of such questions in two seminars. Regarding teacher questions posed with a didactical intention, we first look at the categorical scheme *type of question*, comprising *initial* and *follow-up questions.* As is apparent from Fig. 2, the clinical teachers more often posed initial (67%) as compared to follow-up questions (12%). Regarding *openness of questions*, we found that clinical teachers asked much more *closed* (70%) than *open questions* (28%). Looking at the *cognitive level of teacher questions*, the clinical teachers in our sample asked more than two times as many *reasoning questions* (67%) than *reproduction questions* (31%). One discipline difference emerged in the area of comprehension questions (*p =* 0.05) with physicians from internal medicine having asked more questions of this kind than their colleagues from surgery.

**Fig. 3:**
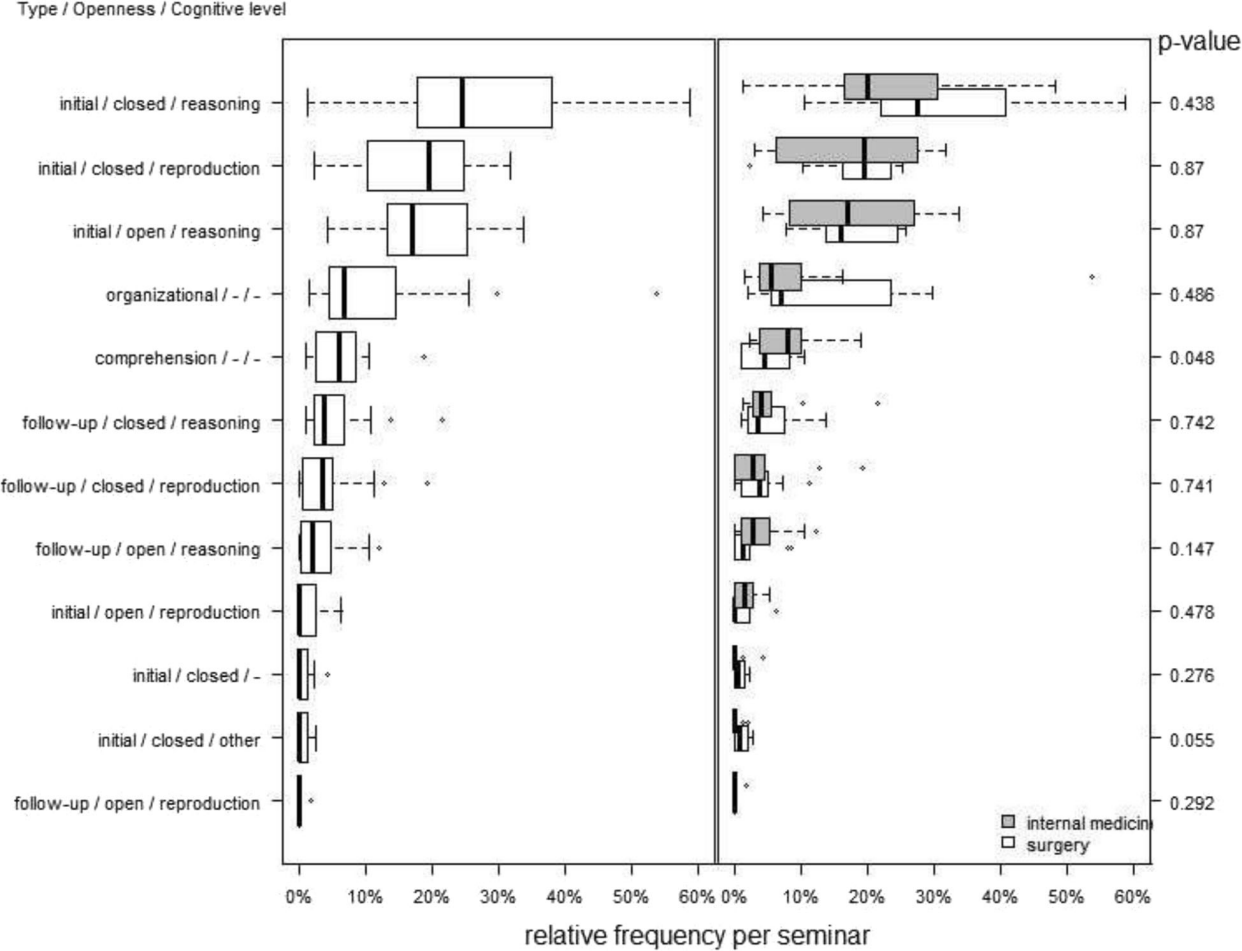
Relative frequencies of question types posed by clinical teachers as percentages of the total amount of questions posed per seminar (left: frequencies across all seminars; right: surgery [white boxplots] and internal medicine [grey boxplots]). Combinations of categories that were not observed in our data or that were observed in less than 25% of the seminars in our sample are not displayed

Figure 3 shows the combinations of categories which were observed, sorted by frequency of occurrence. Overall, three types of didactically relevant questions (= combination of the three didactical categories) dominate the case-based seminars: *initial closed reasoning questions*, *initial closed reproduction questions,* and *initial open reasoning questions*. Regarding medical disciplines, no statistically significant differences emerged.

### RQ2: How do students respond to teacher questions in case-based seminars*?*

**Fig. 4:**
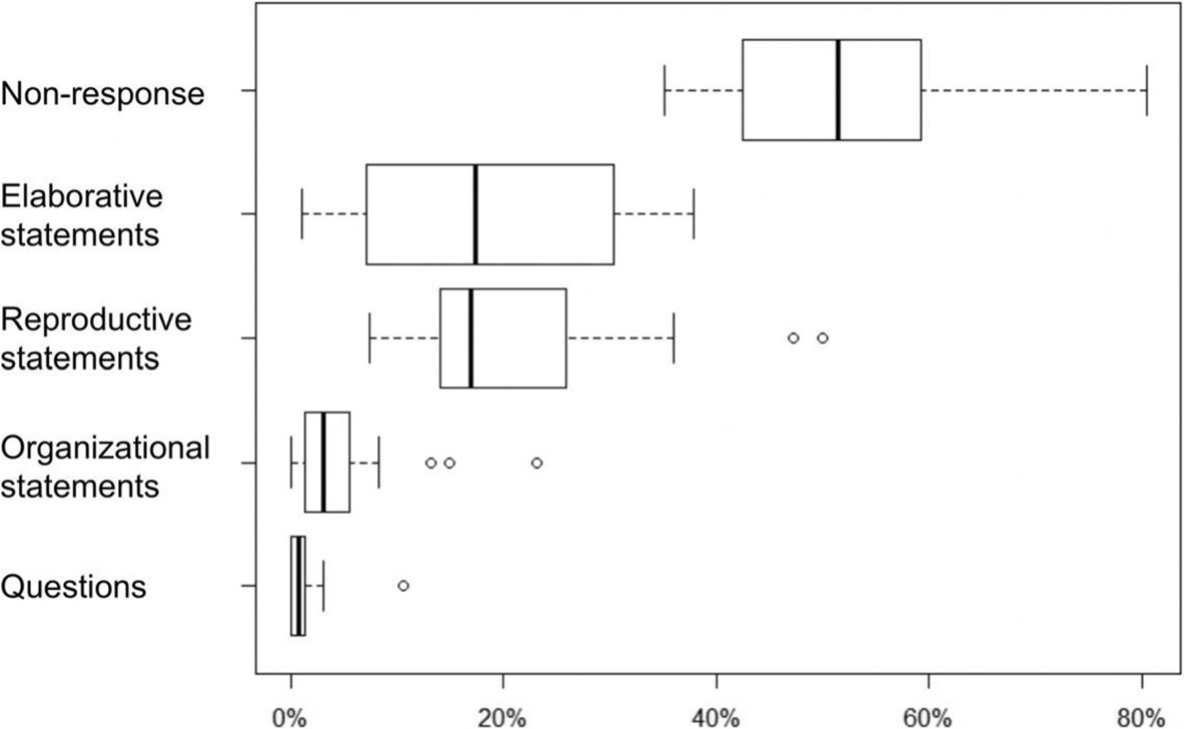
Relative frequencies of different types of student responses / non-responses to clinical teacher questions as percentages of the total amount of student reactions per seminar

Regarding students’ responses to teacher questions, we first look at the number of student responses per seminar and per minute. As is apparent from Table 2, there were some instances in which a teacher question was answered by several students – which led to a higher amount of student responses being observed compared to teacher questions. Figure 4 visualizes the prevalence of different types of student reactions to the questions of clinical teachers (cf. Table 1), i.e. of student non-response, elaborative statements, reproductive statements, organizational statements, and content-related questions.

If looking at the median values reported in Fig. 4, it is apparent that a relatively high degree of teacher questions remained unanswered by students (*MD* = 54%), whereas the amount of elaborative and reproductive responses was almost equal (*MD* = 17%). Very few student responses were concerning organizational matters (3%) or were content-related questions (1%).

### RQ3: How does the type of questions asked by clinical teachers predict reproductive and elaborative responses by students in CBL medical education?

To answer research question 3, we used the evolutionary trees method as explicated in the methods section.

Starting from node 1, it is apparent that the *cognitive level of teacher questions* makes a difference, i.e. whether teacher questions are reproductive or reasoning-oriented. Reproductive questions are associated with a higher amount of reproductive answers (nodes 3 and 4) while reasoning-oriented questions trigger more elaborative answers (nodes 6 and 7). A further difference (node 2) can be found between open and closed reproduction questions. Open vs. closed reproductive questions are associated with a lower degree of reproductive student responses (31% vs. 53%), more elaborative student responses (11% vs. 5%), and a higher student nonresponse (57% vs. 42%). Regarding absolute amounts, closed reproduction questions are posed much more frequent (more than 14 times more often) than open reproduction questions. The right branch originating from node 1, *reasoning questions*, is indirectly connected to nodes 6 and 7 at the bottom. Overall, these are characterized by higher levels of elaborative answers compared to nodes 3 and 4. The highest degree of elaborative student answers—50% in node 6—is associated with follow-up reasoning questions. Node 6 is also characterised by the lowest rate of student non-response in our analysis (30%) and by a comparatively low degree of reproductive student answers (21%). Finally, node 7 is connected to initial reasoning questions and is characterised by a comparatively high degree of student nonresponse (61%). Such questions also trigger elaborative (23%) and reproductive (23%) student answers. As is apparent, node 7 summarises as much as 945 teacher question-student (non-)response sequences. So, the pattern described here is very frequent in our sample of CBL seminars, even more frequently than closed reproduction questions posed by the teachers followed by student (non-)responses. Finally, we found no evidence for a differential functioning of specific question types in the two disciplines.

## Discussion

**Fig. 5:**
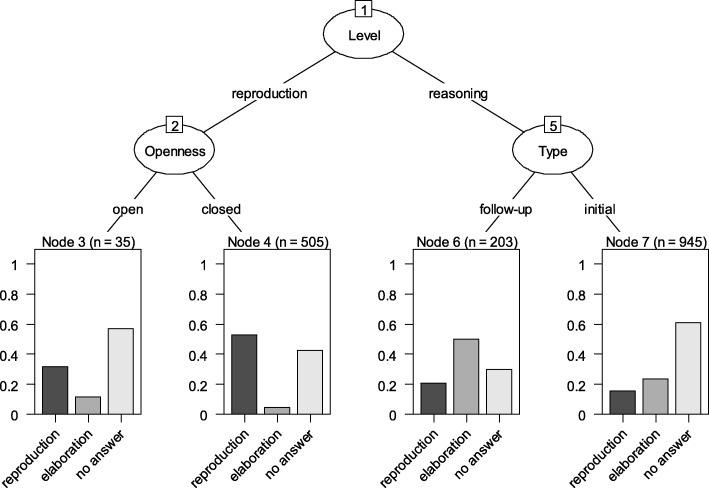
Evolutionary tree diagram describing the relationship between teacher questions and student responses. Oval nodes 1, 2, & 5 and branches in the upper part represent qualities of teacher questions following the coding schemes in Table 1. Nodes 3, 4, 6, & 7 represent types of student responses following a teacher question. Boxes at the bottom display prevalence in percent: dark grey = reproductive student answers, middle grey = elaborative student answers, light grey = no student response. On top of boxes 3, 4, 6, & 7, absolute numbers of cases considered in establishing the respective category are reported

In the present study, we have applied methods of video-based teaching and learning research [21] to a sample of CBL seminars from the disciplines *internal medicine* and *surgery*. Thereby, we have analysed how students’ responses to teacher questions (reproductive and elaborative responses vs. student non-response) depend upon different qualities of questions posed by clinical teachers (type, openness, and cognitive level). In the literature, CBL is mostly described as a format which gives students high degrees of freedom to reflect and to engage in clinical thinking (e.g., [2, 4–6]). On this basis, one could have expected clinical teachers to pose more open than closed and more reasoning than reproductive questions. In our data, we observed the latter (with a reproduction:reasoning questions ratio of about 1:2); unexpectedly, however, clinical teachers used far more closed than open questions (cf. Figs. 2, 3 and 5). This result underscores that it is challenging for clinical teachers in CBL seminars to reach a balance between engaging students in the *reproduction* of relevant basic clinical knowledge and fostering *elaboration/deep reasoning* while focusing upon a specific patient case [4, 6]. If looking at student statements following teacher questions (cf. Fig. 4), however, it is remarkable that the frequencies of *elaborative* and *reproductive* student statements are very similar, overall. In principle, this result confirms the assumption of CBL seminars being characterised by a balanced amount of reproduction and elaboration. However, both distributions show a very broad range. This points towards a substantial heterogeneity regarding the way different teachers practice CBL.

Regarding teacher questions associated with a high degrees of student elaboration, our results show that reasoning questions are powerful in this respect (cf. Fig. 5). Also, follow-up questions (which build upon previous ones) are suitable to move from basic to more complex issues that require students to engage in reasoning and reflection. However, initial reasoning questions (that did not build upon previously discussed subject matter) were used almost five times as often as follow-up reasoning questions (cf. Fig. 2) – despite initial reasoning questions are associated with a high degree of non-response. It seems that initial reasoning questions are challenging for students and hence oftentimes remain unanswered. For clinical teachers, several recommendations can be drawn from this observation: First, they should pose initial questions with reduced complexity to invite students’ active engagement with a specific topic. Second, in order to invite higher-order reflection and elaboration, posing reasoning follow-up questions are useful. As Fig. 5 shows, posing reasoning follow-up questions is not only associated with a high degree of elaborative student answers, but also with low non-response. Third, in order to further decrease student non-response, clinical teachers should give students ample time to understand and reflect upon their questions. Student non-response is a phenomenon which is not well researched in medical education, but which was observed with a high frequency (54% overall) in the present study. Some types of teacher questions – especially open-reproduction and initial-reasoning questions – were associated with relatively high rates of student non-response of up to 60%. Non-response by students is surely not intended by clinical teachers. Especially in the present context, it contradicts descriptions of CBL as an interactive and student-centred format [3, 4].

In theorizing about what makes CBL effective, authors have provided elaborate, yet often quite general, descriptions of the behaviours of both teachers and learners, e.g. “the facilitator orchestrates the discussion to maximize the breadth and depth of the dialogue” or “advances the learning objectives for which the case was designed by formulating a sequence of trigger questions” ([6], p. 835). Regarding students, descriptions are even more general, such as “student discussion and guided inquiry around clinical problems are promoted” [2]. In contrast, the present study contains a fine-grained empirical analysis of the interaction between teachers and learners in CBL. We argue that (video) studies focusing teaching/learning processes on the micro-level are a promising way forward for medical education research (on CBL and in general) for several reasons: On the one hand, the data the research is based upon are generated by researchers in a systematic and closely monitored process. This makes the data less prone to systematic biases [42], like when high inference judgements from teachers/students themselves are collected (e.g., [43]). On the other hand, video studies are valuable to better understand *how clinical teaching actually works* and is practiced by clinical teachers. It can help strengthen a descriptive perspective in clinical teaching research and better understand how certain teaching methods are implemented [44] while not excluding questions about the efficacy of these methods.

### Future research

Based on our processing of the video material, we observed two phenomena which demand further research in the future. First, in many instances, medical teachers simply *out-talked* their students [45]. This means that non-response was connected to very little time being available for students to mentally deliberate upon a teacher’s question and find a potentially suitable answer [13]. Oftentimes, teachers waited only a few seconds before they reformulated their question, posed a different one, or answered their own question. The phenomenon of *teacher wait time* (e.g., [46–49]) is a relevant focus for empirical research in medical education. Earlier research from the school context has shown that if teachers increase post-question wait time, students give longer and more elaborate answers (e.g., [50]). Second, students generally seemed hesitant to answer teacher questions if they were unsure about whether their answer would be correct. So, teachers striving to make students in their seminars more responsive should motivate them to verbalise their thoughts rather than presenting only perfectly correct answers to them. This issue is also connected to the error management behaviour (e.g., [51]) clinical teachers show in their classrooms and to students’ notion of a psychologically safe learning climate during lectures [52, 53].

Regarding the comparison between internal medicine and surgery, we only found minor differences. On this basis, it does not seem justified to conclude that CBL works different in various medical disciplines. However, our findings indicate that there is substantial heterogeneity between how different teachers practice case-based teaching (regardless of discipline). This is apparent from several outcomes; overall, the CBL-teachers posed quite few didactically irrelevant questions. However, we had seven seminars in our sample in which between 20% and just over 50% of the questions posed were focused upon organisational aspects (cf. Table 1). This indicates that the *overall amount* of teacher questions (didactic *and* organisational) was rather small in these seminars – which also means that these seminars were not very interactive, but were more like lectures anchored in example cases with occasional episodes of student-teacher interaction. A critical concern which could be connected to this observation is whether it is legitimate to consider the seminars we observed as valid examples of CBL in the first place. We argue that it can be seen as a strength of the present study to include a broad range of ways in which CBL was implemented (from more teacher-guided to more-student centred). Due to this heterogeneity, our study might not reflect the concept of CBL as discussed in the literature in its purest form. However, we argue that our research has high ecological validity in that it reflects that CBL might be interpreted and practiced by different clinical teachers in very different ways.

### Limitations

A potential limitation is that due to its low inference character, we could only analyse teacher question-student (non-)response dyads. By pursuing a more high-inference analytic strategy, longer passages and several turns of teacher-student interactions could have been analysed. This surely is a promising idea for future research. In the field of empirical educational research, a certain trend away from low-inference analytic approaches in video studies is apparent [23]. A further limitation with regard to the comparison of internal medicine vs. surgery is the small sample of video recorded CBL seminars analysed here. So, our results concerning the comparison of these two broad and diverse medical disciplines should be considered explorative. Furthermore, to be able to compare our results to other dynamic forms of teaching in medical education, video recordings of other formats, such as bedside teaching, should be considered. Unfortunately, no evidence exists which would allow for comparing our results to other didactical formats in medical education, such as problem based learning. So, it seems hard to say whether our results are representative in a broader sense.

## Conclusion

The present study gives detailed insights into associations between clinical teacher questions and medical student responses in CBL. They are relevant for medical teaching practice and professional development. Some concrete recommendations to clinical teachers can be deducted: When seeking to make students elaborate, teachers should primarily ask reasoning questions or follow-up questions. For eliciting reproductive answers from students, closed reproduction question are suitable. To increase student participation, teachers should strive for low student non-response rates. This can be achieved in different ways. Henning et al. [13] recommend open questions which ask for students’ opinions (rather than knowledge). If choosing a knowledge-based angle on a new topic, initial reasoning questions should be avoided. Posing an easy to answer closed reproduction question might be a better idea. Regarding future studies on dialogic teaching in medical education, longitudinal designs that investigate the effects of clinical teacher professional development on teachers’ classroom behaviour could be valuable [54]. In this respect, lowering the rate of student non-response in interactive, small-group seminar settings is a specific challenge apparent from the results of the present study.

## Data Availability

All data (except the original video recordings) and materials are available upon direct request to the authors.

## Abbreviations

CBL: Case-based learning
EKG: Electrocardiogram
IRR: Interrater reliability
MRT: Magnet resonance tomography
SOAP-scheme: Subjective objective assessment plan-scheme
TUM: Technical University of Munich
